# Narcissism Is Associated With Blunted Error‐Related Brain Activity

**DOI:** 10.1111/jopy.70036

**Published:** 2025-12-12

**Authors:** Esther M. Robins, Zhiwei Zhou, Chengli Huang, Douglas J. Angus, Constantine Sedikides, Nicholas J. Kelley

**Affiliations:** ^1^ Centre for Research on Self and Identity, School of Psychology University of Southampton Southampton UK; ^2^ Wenzhou Medical University Wenzhou China; ^3^ School of Psychology Bond University Gold Coast Queensland Australia

**Keywords:** cognitive control, error‐related negativity, event‐related potential, narcissism, self‐regulation

## Abstract

**Objective:**

Narcissism is associated with self‐enhancement and social antagonism, yet its neural underpinnings, particularly in error processing, remain underexplored. Competing theoretical models, such as the mask model and the metacognitive model, offer conflicting hypotheses regarding how narcissism influences early neural responses to errors. We examine whether grandiose agentic narcissism relates to an elevated or blunted error‐related negativity, a neural marker of cognitive control and performance monitoring.

**Method:**

In Study 1 (*N* = 144), participants completed the Eriksen Flanker Task while we recorded their neural responses to errors using electroencephalography. In Study 2 (*N* = 50), participants completed a modified version of the Flanker Task that included an explicit trial‐by‐trial feedback. Participants then completed the Narcissistic Admiration and Rivalry Questionnaire to assess admiration and rivalry narcissism.

**Results:**

Higher admiration and rivalry narcissism were associated with a blunted (less negative) error‐related negativity. These associations held when controlling for the number of errors and were confirmed by an internal meta‐analysis, which showed moderate effect sizes across analytic approaches.

**Conclusion:**

The results are consistent with the metacognitive model of narcissism, showing that grandiose narcissists exhibit reduced neural sensitivity to errors. These findings highlight a potential mechanism through which narcissists resist self‐corrective learning, bolstering their positive self‐views. Blunted error processing may influence decision‐making and behavior across contexts.

Narcissism's contradictory nature has long intrigued researchers and the public. Narcissistic leaders, whether in politics or business, can make decisions that jeopardize the stability of a nation or organization, yet they frequently maintain or even grow their influence (MacDonald 2014; Resick et al. 2009). In interpersonal relationships, narcissists may create tension and conflict, yet they often remain valued or sought‐after companions (Czarna et al. 2022; Giacomin and Jordan 2019). An extensive literature has examined how narcissists can both captivate and disrupt national, institutional, or social spheres. However, much of this literature relies on self‐reports, leaving a gap in understanding the roots of narcissistic cognition and behavior. Personality neuroscience, the study of individual differences using neuroscientific measures, promises to supply the lacking illumination with its objective window into cognitive processes (DeYoung et al. 2022). Specifically, it can examine, with empirical rigor, whether and to what extent narcissists (relative to non‐narcissists) appropriately and adaptively process feedback, particularly when they have made errors. We investigated the relation between narcissism and the earliest neural marker of error processing, the error‐related negativity (ERN).

## 1 Narcissism

Narcissism is a normally distributed personality trait^1^ characterized by egocentric exceptionalism (i.e., perceptions of uniqueness, importance, and superiority) as well as social selfishness (i.e., putting oneself first, usually at others' expense; Sedikides 2021a). The trait manifests in several forms that build on those fundamental characteristics (Krizan and Herlache 2018; Sedikides 2021a). One form is vulnerable narcissism, characterized by introversion, neuroticism, and defensiveness (Miller et al. 2018; Thomaes et al. 2018). However, the most widely studied form and focus of our research is grandiose agentic narcissism, marked by confidence, extraversion, and exhibitionism (Roberts et al. 2018; Sedikides et al. 2004). Grandiose agentic narcissists exalt the self predominantly in the agentic domain, celebrating their intelligence, ambition, vision, and leadership potential (Gebauer and Sedikides 2018; Gebauer et al. 2012). Further, grandiose agentic narcissism can be subdivided into admiration and rivalry (Back et al. 2018, 2013). These two forms share an underlying motivation: to establish and sustain a haughty self‐concept. However, they do so through different strategies. Admirative narcissists pursue and maintain haughtiness via self‐enhancement (i.e., elevating the positivity of their self‐concept), whereas rivalrous narcissists pursue and maintain haughtiness via antagonistic self‐protection (i.e., minimizing the negativity of their self‐concept). That is, admirative narcissists engage in self‐promotion with the anticipation of receiving recognition and acclaim from others, whereas rivalrous narcissists engage in self‐protection aimed at asserting dominance and diminishing the value of others. We focus in this article on admiration and rivalry.

### 1.1 Theoretical Models of Narcissism

Efforts to understand the causes and consequences of narcissists' thoughts, feelings, and behaviors have led to the development of two theoretical models. The mask model of narcissism (hereafter “the mask model”) posits that narcissists maintain a confident external façade to protect against deep‐seated insecurity, leading to hypervigilance and enhanced cognitive control in response to self‐threats. In contrast, the metacognitive model of narcissism (hereafter “the metacognitive model”) proposes that narcissists have a robust inner core that they preserve through cognitive avoidance and distortions. We elaborate on these models and their divergent implications for error processing.

#### 1.1.1 The Mask Model

The mask model was advocated by psychodynamic theorists (Freud 1957; Kernberg 1975; Kohut 1966). According to it, a fundamental discrepancy exists between the narcissists' external and internal selves. Whereas their external self is characterized by a hardened, assured, and radiant facade, their internal self is soft, unstable, and vulnerable. Their puffed‐up persona protects against their profound fragility (Gregg and Sedikides 2010). Due to their fragility, narcissists feel particularly threatened by criticism or negative feedback and react with hypervigilance. For example, high narcissists respond faster to negative and self‐relevant words than low narcissists (Hardaker et al. 2021; Horvath and Morf 2009). Hypervigilance allows narcissists to effectively defend the self and facilitates a stronger sense of cognitive control over outcomes.

#### 1.1.2 The Metacognitive Model

According to the metacognitive model (Carlson 2013; Carlson et al. 2011; Podsiadłowski et al. 2025), narcissists display blunted feedback responses due to a robust inner core that employs cognitive avoidance to dismiss errors and mistakes. In support of this model, narcissists use cognitive distortions to protect their self‐image, ultimately leading to poor learning and decision‐making over time. Specifically, narcissists exhibit reduced counterfactual thinking; that is, they do not critically reflect on what they should have done differently. This lack of self‐examination hinders their ability to learn from their mistakes (Howes et al. 2020). Additionally, narcissists employ cognitive avoidance strategies by shifting blame to external factors rather than accepting personal responsibility. This strategy reinforces their strong inner core and further impedes learning (Howes et al. 2020). Narcissists also display hindsight bias when correct (“I knew it all along”) and reverse hindsight bias when incorrect (“No one could have seen this coming”), allowing them to reframe past events and avoid self‐criticism (Howes et al. 2020). Moreover, when experimentally induced, counterfactual thinking decreases the hindsight bias and improves learning among narcissists (Howes et al. 2020). In a similar vein, grandiose narcissism is associated with metacognitive distortions (i.e., overconfidence) in both prospectively judged memory and retrospectively judged intelligence task performance (Littrell et al. 2024). It is no surprise, then, that narcissists perceive negative feedback as non‐diagnostic and react defensively to it (Kernis and Sun 1994; Zhu and Chen 2015), regard expert advice as non‐credible and distrustful (Kausel et al. 2015; O'Reilly and Hall 2020), and are unlikely to learn from their failures (Campbell et al. 2004; Liu et al. 2019). In all, narcissists resist self‐corrective metacognitive processes, limiting their ability to learn and causing them to repeat mistakes.

#### 1.1.3 Contrasting Implications of the Models

Cognitive control is critical to scholarly understanding of error processing. Errors (e.g., incorrect keypresses) imply that one's behavior violates contextual task demands. Hence, additional resources must be recruited to rectify the situation (i.e., perform a correct keypress). Therefore, errors imply that cognitive control is needed (Meyer and Hajcak 2019). In regard to the mask model, a heightened early neural response to errors reflects grandiose agentic narcissists' capacity to engage cognitive control, demonstrating their hypervigilance to self‐threats from the earliest stages of information processing.

In comparison, the metacognitive model relates differently to cognitive control. According to this model, poorer performance displayed by grandiose narcissists on the abovementioned tasks (Howes et al. 2020; Littrell et al. 2024) may be underpinned by a blunted response to errors (Frank et al. 2005). This reflects their inability to recruit the cognitive resources required to learn from their mistakes.

### 1.2 The Neuroscience of Narcissism

The neuroscience of narcissism is in its infancy. Of the 12,000+ articles that address narcissism (PsycINFO, February 18, 2025), very few (< 1%) have examined its neural underpinnings (Jauk and Kanske 2021). Several of these articles have linked grandiose agentic narcissism to neural indices during a laboratory task. Many of the pertinent studies have relied on functional magnetic resonance imaging and have produced two key neuroanatomical findings. First, grandiose agentic narcissism is associated with greater activity in the anterior cingulate cortex (ACC) when responding to pictures of one's own face (Jauk et al. 2017) and social exclusion (Cascio et al. 2015). Second, grandiose agentic narcissism is associated with reduced activity in the anterior insula when empathizing with others (Fan et al. 2011) and in anticipation of interpersonal touch (Scalabrini et al. 2017). Both the ACC (Ridderinkhof et al. 2004) and anterior insula (Billeke et al. 2020) are key neural substrates of error processing. Therefore, these regions represent a promising starting point for a neuroscientific inquiry into narcissism. Given that the ACC has been more intensively investigated in the context of error and feedback processing, whereas the anterior insula has been implicated across a broader range of psychological phenomena (Gasquoine 2014), we focused on neural signals generated by the ACC, namely, the ERN (Gehring et al. 1993).

We will describe next the relation between narcissism and the ERN. The latter is a negative deflection in the electroencephalography (EEG) waveform that peaks at 50 ms post error and is elicited by errors (i.e., incorrect keypresses) across a variety of contexts—most commonly on speeded reaction time tasks involving social and non‐social stimuli (Suzuki et al. 2020). Errors imply that one's behavior violates contextual task demands. Hence, additional resources must be recruited to rectify the situation, namely, to perform a correct keypress. Hence, errors imply that cognitive control is needed (Meyer and Hajcak 2019). Correct‐related negativity (CRN) is the much smaller deflection following a correct response, also peaking at 50 ms post response. Given that cognitive control is an important function of the ACC (Botvinick 2007), the ACC is widely regarded as the primary neural generator of the ERN (Keil et al. 2010). This relation is strengthened by fMRI and source analysis MEEG (Mathalon et al. 2003; Miltner et al. 2003).

Only one study has examined the relation between narcissism and ERN (Mück et al. 2023). Participants first completed a standardized scale of admiration and rivalry narcissism, the Narcissistic Admiration and Rivalry Questionnaire (NARQ; Back et al. 2013), followed by an initial control block of trials; this was the no self‐threat condition. Next, participants completed an additional block of trials. They were presented with normative performance data and percentile rankings indicating that they performed substantially worse than their peers; this was the self‐threat condition. Rivalry (but not admiration) narcissism was associated with an elevated (more negative) ERN, but not with error positivity. In line with the mask model, direct and explicit self‐threat enhanced cognitive control mechanisms of the ACC manifesting in a larger (more negative) ERN.

### 1.3 Research Overview

The single examination of the association between narcissism and the ERN was limited by small sample size (*N* = 89) and pre‐task personality assessment (Mück et al. 2023). Given that small sample sizes can overestimate effects (Button et al. 2013; Lin 2018), the association between narcissism and an elevated ERN (i.e., neurocognitive support for the mask model) may not be as strong as it appeared to be. Further, assessing a personality trait before an experimental task can prime participants with that trait, influencing subsequent responses and behavior (Nordlund 2009; Weingarten et al. 2016). Specifically, priming can alter the active self‐concept (Skowronski et al. 2010; Wheeler et al. 2014), potentially causing narcissistic individuals to display heightened self‐protection and vigilance to self‐threat. This effect may inadvertently lend artificially support to the mask model by reinforcing expected behaviors rather than capturing genuine, spontaneous responses.

We addressed these limitations across two studies and tested competing hypotheses about the relation between grandiose agentic narcissism and the ERN. First, as per the mask model and supportive findings (Mück et al. 2023), we hypothesize that grandiose narcissism will be associated with an elevated (more negative) ERN, suggesting that narcissists recruit cognitive control resources to hypervigilantly respond to self‐threats in the early stages of information processing. Second, in line with the metacognitive model, we hypothesize that grandiose narcissism will be instead associated with a blunted (less negative) ERN, suggesting impaired learning from mistakes (Frank et al. 2005).

### 1.4 Transparency and Openness

We report how we determined our sample size, all data exclusions, all manipulations, and all measures in the study, and we follow journal article reporting standards (Appelbaum et al. 2018). Our studies were approved by the Psychology Ethics Committee, University of Southampton (Protocol Number 68828). All research materials, data, and analysis code are available at: https://osf.io/gubyp/?view_only=1d4dfd3202ac4184bcffe7f047cc9432.

## 2 Study 1

In Study 1, we examined the association between grandiose agentic narcissism and early neural responses to errors (ERN) using the Eriksen Flanker Task while EEG was recorded, followed by a battery of questionnaires including the NARQ (Back et al. 2013).

### 2.1 Method

#### 2.1.1 Participants and Procedure

We recruited 161 University of Southampton undergraduate students, who participated in exchange for course credit. We continued to test participants until the end of the academic year. After removing participants based on the criteria below, the final sample was *N* = 144. It conferred 80% power to detect effects as small as *r* = .23 (Faul et al. 2007). Participants were 18–46 years of age (*M* = 19.61, SD = 2.93). They identified as female (*n* = 116, 80.56%), male (*n* = 27, 18.75%), or did not report their gender (*n* = 1, 0.69%). To test our hypotheses, we implemented the Eriksen Flanker Task (Eriksen and Eriksen 1974) while EEG was recorded. Finally, we administered the NARQ at the end of the session, nesting it within a larger questionnaire battery to disguise the study's intent.

The evidence surrounding an acceptable number of trials to produce a reliable ERN is inconsistent. Some researchers suggest that as few as six trials are needed (Olvet and Hajcak 2009), whereas others recommend 16 trials (Clayson 2020). Following the approach of Meyer et al. (2013), we examined how the ERN amplitude changes as a function of cumulative errors. We isolated the first 2, 4, 6, 8, 10, 12, 14, 16, 18, and 20 errors and then correlated the ERN for each of these bins of errors with the grand average for each participant. For Fz, where the ERN was maximal for our data, correlations were > 0.80 as of six errors. Thus, we adopted six errors as our cut‐off for a reliable ERN and excluded participants who produced less than six errors. We excluded an additional participant who made errors on over 90% of trials.

#### 2.1.2 Eriksen Flanker Task

The Eriksen Flanker Task is a speeded reaction time task commonly used to evoke an ERN. On each trial, five horizontal arrowheads are presented for 200 ms, and participants respond as accurately and quickly as possible to the direction of the middle arrowhead by pressing the left or right arrow key on the keyboard. On congruent trials, all five arrowheads point in the same direction (>>>>> or <<<<<). On incongruent trials, the middle arrowhead points in the opposite direction of the flanking arrowheads (<<><< or >><>>). Participants completed four self‐paced blocks of 76 trials (304 trials), with an equal proportion of congruent and incongruent trials. Psychometric studies recommend the Flanker Task for studies on individual differences because it yields a reliable ERN (*α* = 0.80 to 0.90) with few (6–14) errors (Meyer et al. 2013). Based on established norms (Imburgio et al. 2020), we expected participants to commit errors on 11% of trials and thus produce 33 errors over 304 trials. As the ERN achieves good reliability with few errors, 304 trials are sufficient to yield a reliable ERN. Immediately following the Flanker Task, participants completed two additional EEG tasks unrelated to the current investigation. Subsequently, they filled out a battery of individual difference measures, including narcissism (see below).

#### 2.1.3 EEG Recording and Data Reduction

We recorded EEG from 32 active Ag/AgCl electrodes, arranged according to the international 10–20 system. We sampled signals at 500 Hz using an ActiChamp amplifier, with FCz and Cz serving as the ground and reference, respectively. Further, we kept electrode impedances below 10 kΩ.

We preprocessed EEG data in Python 3.12.2 4, using MNE 1.8.0. First, we downsampled the data to 250 Hz, referenced them to the average of the mastoids (TP9 and TP10), and bandpass filtered them between 0.1 and 30 Hz. We used Independent Component Analysis (Jung et al. 2000), followed by ICLabel (Pion‐Tonachini et al. 2019) to identify and remove components classified as non‐neural in origin. We then epoched the data to the −500 ms before to 500 ms after responses, and baseline corrected to the −400 to −200 ms before each response. We excluded epochs with voltage differences of ±200 μV from further analysis. We subsequently averaged retained epochs for correct and incorrect responses. Also, we excluded participants with fewer than six artifact‐free trials for either correct or incorrect responses. Retained participants had between 286 and 304 trials (*M* = 303.17, SD = 2.63) across both trial types (correct + incorrect). The number of correct trials ranged from 178 to 298 (*M* = 281.90, SD = 18.42), and the number of incorrect trials ranged from 6 to 126 (*M* = 21.27, SD = 18.27).

Consistent with earlier research (Meyer et al. 2013), we estimated the ERN using the mean amplitude 0–50 ms post‐response for both incorrect (ERN) and correct (CRN) responses. We also created a difference wave (incorrect—correct) and used this to estimate the ΔERN. We extracted estimates from Fz and Cz.

#### 2.1.4 Narcissistic Admiration and Rivalry Questionnaire

The NARQ assesses grandiose agentic narcissism through a two‐subscale approach. Participants indicated their level of agreement with 18 statements from 1 (*not agree at all*) to 6 (*agree completely*). Nine items each assess admirative (e.g., “I deserve to be seen as a great Personality”) and rivalrous (e.g., “I secretly take pleasure in the failure of my rivals”) forms of grandiose agentic narcissism. The average score on the admiration subscale ranged from 1.22 to 5.33 (*M* = 3.22, SD = 0.82, *α* = 0.83), and the average score on the rivalry subscale ranged from 1.00 to 4.56 (*M* = 1.70, SD = 0.71, *α* = 0.86). Average NARQ composite scores ranged from 1.28 to 4.22 (*M* = 2.46, SD = 0.59, *α* = 0.84).

### 2.2 Results

#### 2.2.1 Preliminary Analyses

We conducted a 2 (Site: Cz, Fz) × 2 (Response: correct, error) within‐subjects Analysis of Variance (ANOVA) to examine brain activity 0 to 50 ms post‐response. The response main effect was significant: Error responses (*M* = −3.3 μV, SE = 0.43) were more negative than correct responses (*M* = 2.65 μV, SE = 0.30), *F*(1, 143) = 180.62, *p* < 0.001, partial *η* ^2^ = 0.558. The site main effect was also significant: Responses were more negative at Fz (*M* = −1.15 μV, SE = 0.30) than at Cz (*M* = 0.48 μV, SE = 0.33), *F*(1, 143) = 54.83, *p* < 0.001, partial *η* ^2^ = 0.277.

**FIGURE 1:**
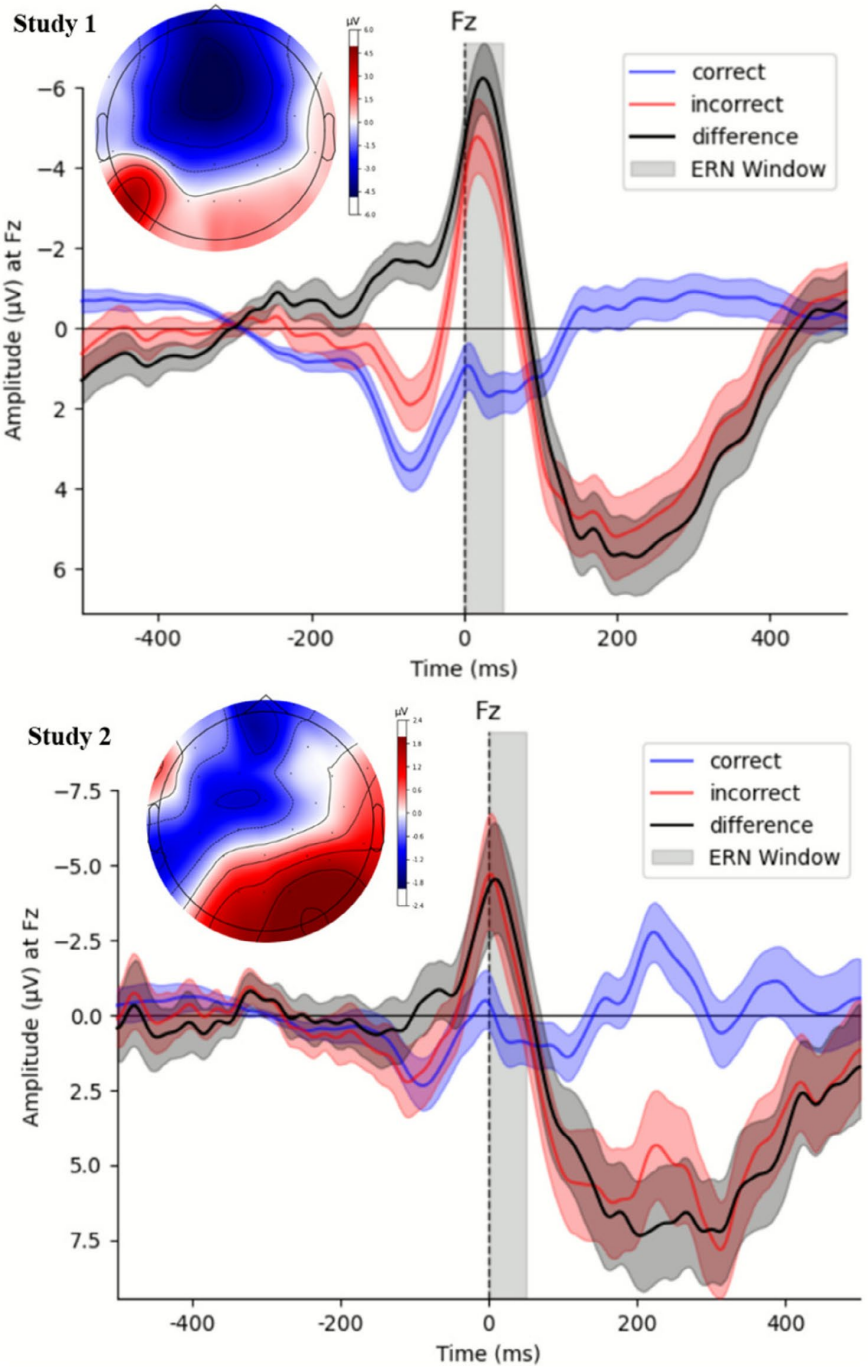
Grand average waveform and topographic map of the error‐related negativity.

These main effects were qualified by a Site × Response interaction, *F*(1, 143) = 7.01, *p* = 0.009, partial *η* ^2^ = 0.047. We broke it down by examining the simple main effect of site separately for correct and error responses. Error responses were larger (more negative) at Fz (*M* = −3.92 μV, SE = 0.45) than at Cz (*M* = −2.70 μV, SE = 0.47, *p* < 0.001), *F*(1, 143) = 13.54, *p* < 0.001, partial *η* ^2^ = 0.086. Likewise, correct responses were larger (i.e., more negative) at Fz (*M* = 1.62 μV, SE = 0.29) than at Cz (*M* = 3.67 μV, SE = 0.34, *p* < 0.001), *F*(1, 143) = 119.68, *p* < 0.001, partial *η* ^2^ = 0.456. We illustrate these results in Figure 1.

#### 2.2.2 Associations Between Narcissism and Error‐Related Brain Activity

Given that ERN was strongest over Fz, we focused our analysis on this site.

**FIGURE 2:**
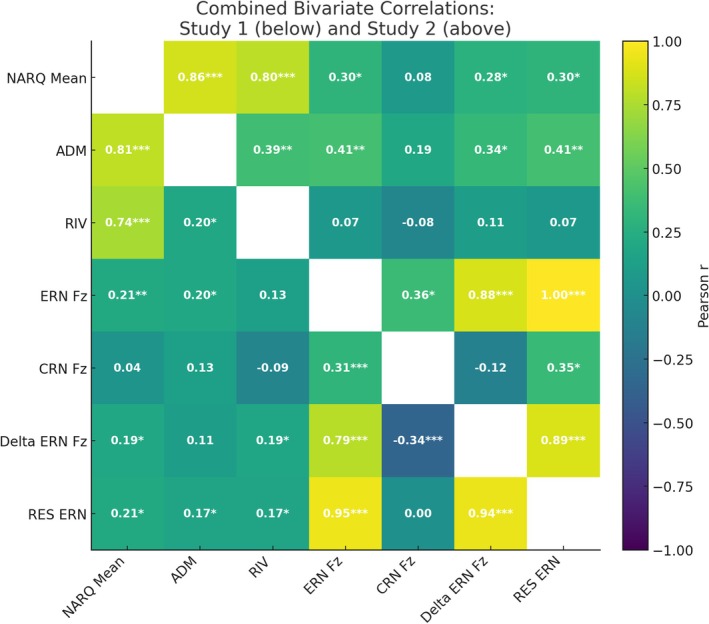
Bivariate correlations between error‐related brain activity and narcissism. Pearson's correlations from Study 1 are presented below the diagonal; correlations from Study 2 are presented above the diagonal. ADM, admiration subscale; CRN, correct‐related negativity; ERN, error‐related negativity; NARQ, Narcissistic Admiration and Rivalry Questionnaire Total Score; rERN, residual ERN; RIV, rivalry subscale; ΔERN, difference wave. *p < 0.05, **p < 0.01, ***p < 0.001.

We used bivariate correlations to examine associations between narcissism, ERN, CRN, and ΔERN (Figure 2; below the diagonal). A larger (more negative) ERN was associated with lower overall narcissism (NARQ), *r* = .215, *p* = 0.010. This association did not differ for admirative (*r* = .198, *p* = 0.015) compared with rivalrous (*r* = .131, *p* = 0.117) narcissism, *Z* = 0.64, *p* = 0.521. Sensitivity power analyses in G*Power (Faul et al. 2007) indicated that we had 80% power to detect correlations as small as *r* = .23. Thus, we were just under the threshold for the narcissism total score and admirative narcissism. Overall narcissism, admirative narcissism, and rivalrous narcissism were unrelated to CRN.

To isolate the ERN from overlapping brain activity, we used a difference wave procedure (Kappenman and Luck 2012; Luck 2014) and calculated the ΔERN as the difference between the ERN and the CRN (ERN—CRN). Results revealed that a larger (more negative) ΔERN was associated with higher overall narcissism, *r* = .188, *p* = 0.024. This association did not differ for rivalrous (*r* = .187, *p* = 0.025) compared with admirative (*r* = .110, *p* = 0.189) narcissism, *Z* = 0.74, *p* = 0.462. Sensitivity power analyses in G*Power (Faul et al. 2007) indicated that we had 80% power to detect correlations as small as *r* = .23. Thus, we were under powered for narcissism analyses with the ΔERN.

As an alternative to the ΔERN, researchers have used a residualized ERN approach (Clayson et al. 2021). The residualized ERN is the unstandardized residual obtained by regressing the ERN on the CRN, capturing variance in the ERN that is not attributable to the CRN. The same logic applies in reverse: regressing the CRN on the ERN yields a residualized CRN, which reflects variance in the CRN not shared with the ERN. Compared with a simple difference score, this approach better isolates the unique contribution of each component by accounting for their shared variance (Meyer 2016; Sandre et al. 2020). A larger (more negative) residual ERN was associated with higher overall narcissism, *r* = .214, *p* = 0.010. This association did not differ for rivalrous (*r* = .166, *p* = 0.046) compared with admirative (*r* = .165, *p* = 0.048) narcissism, *Z* = 0.01, *p* = 0.992. Sensitivity power analyses in G*Power (Faul et al. 2007) indicated that we had 80% power to detect correlations as small as *r* = .23. Thus, we were slightly under the threshold for analyses with the residual ERN.

**FIGURE 3:**
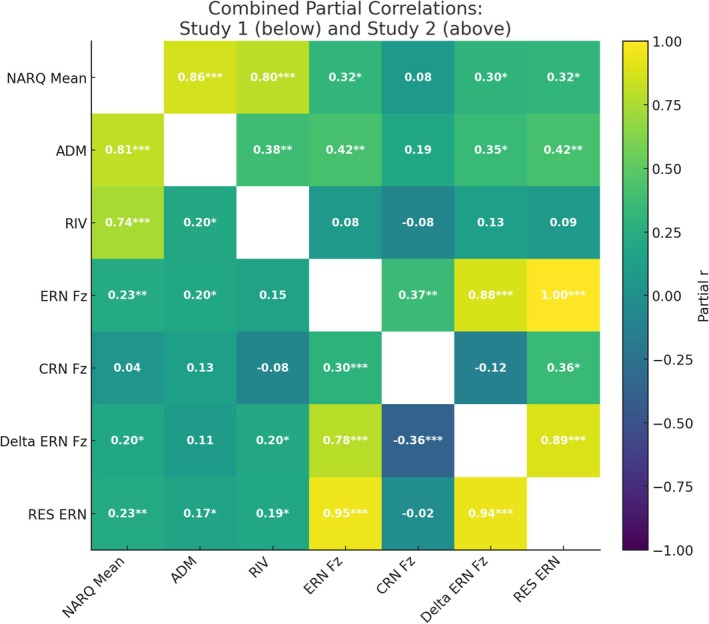
Partial correlations between error‐related brain activity and narcissism. Partial correlations from Study 1 are presented below the diagonal; correlations from Study 2 are presented above the diagonal. ADM, admiration subscale; CRN, correct‐related negativity; ERN, error‐related negativity; NARQ, Narcissistic Admiration and Rivalry Questionnaire Total Score; RIV, rivalry subscale; ΔERN, difference wave. *p < 0.05, **p < 0.01, ***p < 0.001.

The number of incorrect trials was unrelated to any form of narcissism or CRN (*r*s < .10, *p*s > 0.20). Committing more errors was associated with a smaller (less negative) ERN (*r* = .208, *p* = 0.012) and a smaller residual ERN (*r* = .191, *p* = 0.022). Given the associations between the number of errors and ERN/residual ERN, we repeated the correlational analyses controlling for the number of errors (Figure 3). When controlling for the number of errors, a larger (more negative) ERN was associated with lower overall narcissism (NARQ), *r* = .229, *p* = 0.006. This association did not differ for admirative (*r* = .198, *p* = 0.018) compared with rivalrous (*r* = .154, *p* = 0.066) narcissism, *Z* = 0.42, *p* = 0.672. Additionally, when controlling for the number of errors, a larger (more negative) ΔERN was associated with higher overall narcissism, *r* = .197, *p* = 0.018. This association did not differ for rivalrous (*r* = .204, *p* = 0.015) compared with admirative (*r* = .109, *p* = 0.196) narcissism, *Z* = 0.91, *p* = 0.362. Likewise, when controlling for the number of errors, a larger (more negative) residual ERN was associated with higher overall narcissism, *r* = .226, *p* = 0.007. This association did not differ for rivalrous (*r* = .188, *p* = 0.025) compared with admirative (*r* = .165, *p* = 0.049) narcissism, *Z* = 0.22, *p* = 0.825.

We conducted a sensitivity power analysis using G*Power 3.1 (Faul et al. 2009) to determine the minimum detectable effect size for the unique association between narcissism and residual ERN, controlling for the number of errors. The analysis was based on an *F* test using the “linear multiple regression: fixed model, *R* ^2^ increase” option, with *α* = 0.05, power = 0.80, and a total sample size of *N* = 144. We specified narcissism as the predictor, with the number of errors entered as a covariate (i.e., a total of two predictors in the model). Study 1 was powered to detect a minimum effect size of *f* ^2^ = 0.055, corresponding to a partial correlation of approximately *r* = .23. Thus, the analyses controlling for the number of errors were sufficiently powered to detect the observed association between overall narcissism and residual ERN, supporting the reliability of this finding.

#### 2.2.3 Bayesian Analyses

We also repeated the analyses above taking a Bayesian approach in JASP 0.19.3.0 (JASP Team 2025). We applied Lee and Wagenmakers' (2013) classification scheme. Bayes factors between 1 and 3 are considered anecdotal evidence supporting the alternative hypothesis, Bayes factors between 3 and 10 are considered moderate evidence supporting the alternative hypothesis, and Bayes factors > 10 are considered strong evidence supporting the alternative hypothesis. Likewise, Bayes factors between 1/3 and 1 are considered anecdotal evidence supporting the null hypothesis, Bayes factors between 1/10 and 1/3 are considered moderate evidence supporting the null hypothesis, and Bayes factors < 1/10 are considered strong evidence supporting the null hypothesis.

Our finding that a larger (more negative) ERN was associated with lower overall narcissism (*r* = .215, *p* = 0.010) represents anecdotal evidence supporting the alternative hypothesis (BF_10_ = 2.85). The same pattern emerged for the ΔERN (BF_10_ = 1.33) and residualized ERN (BF_10_ = 2.77). Associations with admirative narcissism also represented anecdotal evidence supporting the alternative hypothesis using the ERN (BF_10_ = 1.70) but moderate evidence supporting the null hypothesis using the ΔERN (BF_10_ = 0.25) and anecdotal evidence supporting the null hypothesis using the residualized ERN (BF_10_ = 0.73). Associations with rivalrous narcissism represented anecdotal evidence supporting the null hypothesis using the ERN (BF_10_ = 0.35) but anecdotal evidence supporting the alternative hypothesis using the ΔERN (BF_10_ = 1.26) and anecdotal evidence supporting the null hypothesis using the residualized ERN (BF_10_ = 0.75).

We also repeated our analyses controlling for the number of errors using Bayesian linear regression. Relative to a null model including the number of errors, a model including overall narcissism represented moderate evidence in support of the alternative hypothesis for the ERN (BF_10_ = 7.74), ΔERN (BF_10_ = 3.18), and residualized ERN (BF_10_ = 7.23). A model including admirative narcissism represented moderate evidence in support of the alternative hypothesis for the ERN (BF_10_ = 3.19) but anecdotal evidence supporting the null hypothesis using the ΔERN (BF_10_ = 0.54) and anecdotal evidence supporting the alternative hypothesis using residualized ERN (BF_10_ = 1.45). A model including rivalrous narcissism represented anecdotal evidence in support of the alternative hypothesis for the ERN (BF_10_ = 1.13) and residualized ERN (BF_10_ = 2.46) but moderate evidence using the ΔERN (BF_10_ = 3.80).

### 2.3 Discussion

Contrary to the mask model, we found in Study 1 that higher grandiose narcissism was associated with a smaller (less negative) ERN, suggesting blunted early neural sensitivity to errors. This effect held across both admiration and rivalry facets, remained when controlling for the number of errors, and was specific to error‐related activity. Although the initial zero‐order correlations may have been underpowered, the analyses controlling for the number of errors were sufficiently powered to detect the observed association between overall narcissism and the residual ERN, supporting the reliability of this finding. Bayesian analyses provided converging support: at the zero‐order level, Bayes factors indicated anecdotal evidence for associations between overall narcissism and the ERN, ΔERN, and residualized ERN, and, when controlling for the number of errors, Bayesian regression models indicated moderate evidence for the contribution of overall narcissism to each ERN measure. Associations for the narcissism facets showed mixed levels of Bayesian evidence across ERN measures. The results align with the metacognitive model: narcissists avoid negative performance cues. However, Study 1 involved only internal error monitoring. Next, we tested whether these effects persist when participants receive feedback after each trial.

## 3 Study 2

In Study 2, we sought to conceptually replicate the Study 1 findings using a similar task structure but with a key modification: participants received a trial‐by‐trial feedback indicating whether their responses were correct or incorrect. This addition enabled us to test whether the blunted neural response to errors observed in Study 1 extends to situations in which performance failures are clearly signaled by external evaluative cues.

### 3.1 Method

#### 3.1.1 Participants and Procedure

We recruited 120 University of Southampton undergraduate students, who participated in exchange for course credit. We excluded participants for making no errors on the Flanker task (*n* = 28),^2^ and returning either non‐useable data after artifact rejection (*n* = 7) or no narcissism data (*n* = 1), leaving a total of 84 participants at this stage. As in Study 1, we examined how the ERN amplitude changes as a function of cumulative errors (Meyer et al. 2013). We isolated the first 2, 4, 6, 8, 10, 12, 14, 16, 18, and 20 errors, and then correlated the ERN for each of these bins of errors with the grand average for each participant. For Fz, where the ERN was maximal for our data, correlations were > 0.80 as of two errors (80 participants' data met this threshold). However, to remain consistent with Study 1 and minimum recommended thresholds in the literature (Olvet and Hajcak 2009), we maintained six errors as our cut‐off for a reliable ERN and excluded participants with fewer (*n* = 34), leaving data from 50 participants for analyses.^3^

Participants were 18–43 years of age (*M* = 19.40, SD = 3.59). They were identified as female (*n* = 40, 80.00%), male (*n* = 8, 16.00%), or did not report their gender (*n* = 2, 2.00%). We implemented a version of the Eriksen Flanker Task (Eriksen and Eriksen 1974) that included explicit trial‐wise feedback while EEG was recorded (Llerena et al. 2016).^4^ Finally, we administered the NARQ at the end of the session and nested it within a larger questionnaire battery to conceal the study's purpose.

#### 3.1.2 Eriksen Flanker Task

The Eriksen Flanker Task was identical to that of Study 1, with the exception that participants received accuracy feedback on each trial (“Correct” or “Incorrect”). In one condition, they were informed that the feedback was generated by two researchers in another room and displayed in a handwriting‐style font (‘Shorelines Script Bold’). In the other condition, they were informed that the feedback was computer‐generated and shown in Arial. All other on‐screen instructions, presented in Open Sans, were identical to those of Study 1.

#### 3.1.3 EEG Recording and Data Reduction

We used identical procedures to Study 1's. We recorded EEG from 32 active Ag/AgCl electrodes, arranged according to the international 10–20 system. We sampled signals at 500 Hz using an ActiChamp amplifier, with FCz and Cz serving as the ground and reference, respectively. Further, we kept electrode impedances below 10 kΩ.

We preprocessed EEG data in Python 3.12.2 4, using MNE 1.8.0. First, we downsampled the data to 250 Hz, referenced them to the average of the mastoids (TP9 and TP10), and bandpass filtered them between 0.1 and 30 Hz. We implemented Independent Component Analysis (Jung et al. 2000), followed by ICLabel (Pion‐Tonachini et al. 2019) to identify and remove components classified as non‐neural in origin. We then epoched the data to the −500 ms before to 500 ms after responses and baseline corrected to the −400 to −200 ms before each response. We excluded epochs with voltage differences of ±200 μV from further analysis. Afterwards, we averaged retained epochs for correct and incorrect responses.

Retained participants had between 216 and 304 trials (*M* = 283.00, SD = 34.08) across both trial types (correct + incorrect). The number of correct trials ranged from 206 to 298 (*M* = 267.88, SD = 34.17), and the number of incorrect trials ranged from 6 to 87 (*M* = 15.12, SD = 13.92). As in Study 1, we estimated the ERN using the mean amplitude 0–50 ms post‐response for both incorrect (ERN) and correct (CRN) responses. We also created a difference wave (incorrect—correct) to estimate the ΔERN and a residualized ERN/CRN.

#### 3.1.4 Narcissistic Admiration and Rivalry Questionnaire

The average score on NARQ's admiration subscale ranged from 1.00 to 4.67 (*M* = 2.62, SD = 0.79, *α* = 0.84), and the average score on the rivalry subscale ranged from 1.00 to 4.56 (*M* = 2.35, SD = 0.68, *α* = 0.73). Average composite scores ranged from 1.00 to 4.06 (*M* = 2.48, SD = 0.68, *α* = 0.87).

### 3.2 Results

#### 3.2.1 Preliminary Analyses

First, we conducted a 2 (Site: Cz, Fz) × 2 (Response: correct, error) within‐subjects ANOVA to examine brain activity 0 to 50 ms post‐response. We obtained a main effect of response: Error responses (*M* = −2.16 μV, SE = 0.92) were more negative than correct responses (*M* = 1.57 μV, SE = 0.46), *F*(1, 49) = 20.93, *p* < 0.001, partial *η* ^2^ = 0.299. Also, we obtained a main effect of site: responses were more negative at Fz (*M* = −1.21 μV, SE = 0.58) than at Cz (*M* = 0.62 μV, SE = 0.67), *F*(1, 49) = 32.51, *p* < 0.001, partial *η* ^2^ = 0.399.

The Site × Response interaction was not significant, *F*(1, 49) = 1.71, *p* = 0.198, partial *η* ^2^ = 0.034. Yet, we broke down the interaction to be consistent with Study 1. Error responses were larger (more negative) at Fz (*M* = −2.93 μV, SE = 0.92) than at Cz (*M* = −1.39 μV, SE = 0.97), *F*(1, 49) = 12.91, *p* < 0.001, partial *η* ^2^ = 0.21. Likewise, correct responses were larger (i.e., more negative) at Fz (*M* = 0.52 μV, SE = 0.44) than at Cz (*M* = 2.63 μV, SE = 0.54), *F*(1, 49) = 37.41, *p* < 0.001, partial *η* ^2^ = 0.43. We illustrate these results in Figure 1.

#### 3.2.2 Associations Between Narcissism and Error‐Related Brain Activity

Given that the ERN was strongest at Fz, analyses focused on this site (Figure 2, above the diagonal). A lower (more negative) ERN was associated with lower overall narcissism (*r* = .30, *p* = 0.033) and stronger for admiration (*r* = .41, *p* = 0.003) and rivalry (*r* = .07, *p* = 0.642), *Z* = 2.29, *p* < 0.026. Narcissism was unrelated to the CRN. As in Study 1, we used both ΔERN and residual ERN scores to isolate error‐specific activity. The ΔERN was associated with higher overall narcissism (*r* = .280, *p* = 0.049), and was larger for admiration (*r* = .335, *p* = 0.017) than rivalry (*r* = .112, *p* = 0.440), albeit not significantly so, *Z* = 1.44, *p* = 0.150. The residual was associated with higher overall narcissism (*r* = .302, *p* = 0.033), and was larger for admiration (*r* = .405, *p* = 0.004) than rivalry (*r* = .189, *p* = 0.188), albeit not significantly so, *Z* = 1.44, *p* = 0.151. Sensitivity analyses indicated that we had 80% power to detect effects of *r* = .38, so these analyses were underpowered to detect the moderately sized effects we observed for overall narcissism (*r* = ~.30), but the associations for admirative narcissism were appropriately powered. The number of incorrect trials was unrelated to any form of narcissism CRN, ERN, ΔERN, or residual ERN (*rs* < |0.15|, *ps* > 0.30). To maintain consistency with Study 1, we repeated the correlational analyses, controlling for the number of errors (Figure 3). When controlling for the number of errors, a larger (more negative) ERN was associated with lower overall narcissism (NARQ), *r* = .320, *p* = 0.025. This association was stronger for admirative (*r* = .418, *p* = 0.003) compared with rivalrous (*r* = .084, *p* = 0.568) narcissism, *Z* = 2.20, *p* = 0.028. When controlling for the number of errors, ΔERN was associated with higher overall narcissism (*r* = .301, *p* = 0.036), and was larger for admiration (*r* = .349, *p* = 0.014) than rivalry (*r* = .132, *p* = 0.367), albeit not significantly so, *Z* = 1.40, *p* = 0.160. Likewise, when controlling for the number of errors, a larger (more negative) residual ERN was associated with higher overall narcissism, *r* = .320, *p* = 0.025. This association was larger for admirative (*r* = .418, *p* = 0.003) compared with rivalrous (*r* = .085, *p* = 0.563) narcissism, *Z* = 2.19, *p* = 0.028.

We conducted a sensitivity power analysis using G*Power 3.1 (Faul et al. 2007) to determine the minimum detectable effect size for the unique association between narcissism and residual ERN, controlling for the number of errors. The analysis was based on an *F* test using the “linear multiple regression: fixed model, *R* ^2^ increase” option, with *α* = 0.05, power = 0.80, and a total sample size of *N* = 50. We specified narcissism as the predictor, entering the number of errors as a covariate (i.e., a total of two predictors in the model). The analysis indicated that the study was powered to detect a minimum effect size of *f* ^2^ = 0.164, corresponding to a partial correlation of approximately *r* = .37. This reflects the smallest effect for the unique contribution of narcissism to residual ERN, beyond variance accounted for by the number of errors, that the study could reliably detect with 80% power. Thus, the analyses controlling for the number of errors were sufficiently powered to detect the observed association between overall narcissism and both ERN and residual ERN, supporting the reliability of this finding.

#### 3.2.3 Bayesian Analyses

Our finding that higher overall narcissism was associated with a blunted (less negative) ERN (BF_10_ = 1.60), ΔERN (BF_10_ = 1.60), and residualized ERN (BF_10_ = 1.60) reflects anecdotal evidence supporting the alternative hypothesis. Our finding that higher admirative narcissism was associated with a blunted ERN (BF_10_ = 11.16) and residualized ERN (BF_10_ = 11.05) reflects strong evidence supporting the alternative hypothesis, whereas analyses with ΔERN (BF_10_ = 2.75) reflect anecdotal evidence supporting the alternative hypothesis. The associations between rivalrous narcissism and ERN (BF_10_ = 0.196), rivalrous narcissism and ΔERN (BF_10_ = 0.236), and rivalrous narcissism and residualized ERN (BF_10_ = 0.197) reflect moderate evidence supporting the null hypothesis.

We repeated our analyses, controlling for the number of errors, using Bayesian linear regression. Relative to a null model including the number of errors, a model including overall narcissism represented moderate evidence in support of the alternative hypothesis for the ERN (BF_10_ = 3.04) and residualized ERN (BF_10_ = 3.06) but anecdotal evidence in support of the alternative hypothesis for ΔERN (BF_10_ = 2.36). A model including admirative narcissism represented strong evidence in support of the alternative hypothesis for the ERN (BF_10_ = 15.91) and residualized ERN (BF_10_ = 15.79) but moderate evidence in support of the alternative hypothesis using the ΔERN (BF_10_ = 4.68). A model including rivalrous narcissism represented anecdotal evidence in support of the null hypothesis for the ERN (BF_10_ = 0.45), ΔERN (BF_10_ = 0.54), and residualized ERN (BF_10_ = 0.45).

### 3.3 Discussion

In Study 2, we conceptually replicated the Study 1 findings using a modified Flanker task that included explicit trial‐by‐trial feedback. Even in the presence of external evaluative cues, higher grandiose narcissism was associated with a smaller (less negative) ERN, suggesting blunted early neural sensitivity to performance failures. This association was evident for both raw and residualized ERN scores and was stronger for admiration than rivalry, though not significantly so. Although bivariate correlations were underpowered to detect effects of the observed magnitude, analyses controlling for the number of errors were sufficiently powered, reinforcing the reliability of the association between narcissism and diminished error‐related neural activity. Bayesian analyses were consistent with this pattern: zero‐order models indicated anecdotal evidence for overall narcissism and strong evidence for admiration, with moderate evidence supporting the null for rivalry, and Bayesian models controlling for the number of errors provided moderate evidence for overall narcissism and strong to moderate evidence for admiration, with anecdotal evidence supporting the null for rivalry. We proceeded with an internal meta‐analysis.

## 4 Internal Meta‐Analysis

To provide a more precise estimate of the associations between narcissism and error‐related brain activity, we conducted an internal meta‐analysis across Studies 1 and 2 using a fixed‐effects approach (Goh et al. 2016). We report this meta‐analysis as a transparent summary of all studies conducted for this project, none of which were omitted or selectively reported, while acknowledging the broader methodological concerns associated with internal meta‐analytic techniques (Ueno et al. 2016; Vosgerau et al. 2019). We extracted effect sizes for overall narcissism, admiration, and rivalry across three indices of error processing: the ERN, ΔERN, and residual ERN. This allowed us to evaluate the consistency and magnitude of these associations across studies and analytic approaches.

The overall weighted mean associations between overall narcissism and error‐related brain activity were significant across all ERN indices: ERN (*r* = .237, SE = 0.073, *Z* = 3.32, 95% CI [0.098, 0.367]), ΔERN (*r* = .217, SE = 0.073, *Z* = 3.02, 95% CI [0.077, 0.348]), and residual ERN (*r* = .232, SE = 0.073, *Z* = 3.24, 95% CI [0.093, 0.362]). The overall weighted mean associations between admirative narcissism and error‐related brain activity were significant across all ERN indices: ERN (*r* = .254, SE = 0.073, *Z* = 3.56, 95% CI [0.116, 0.382]), ΔERN (*r* = .168, SE = 0.073, *Z* = 2.80, 95% CI [0.027, 0.303]), and residual ERN (*r* = .228, SE = 0.073, *Z* = 3.19, 95% CI [0.089, 0.359]). Also, the overall weighted mean associations between rivalrous narcissism and error‐related brain activity were smaller across all ERN indices: ERN (*r* = .116, SE = 0.073, *Z* = 1.60, 95% CI [−0.027, 0.254]), ΔERN (*r* = .168, SE = 0.073, *Z* = 2.33, 95% CI [0.027, 0.303]), and residual ERN (*r* = .173, SE = 0.073, *Z* = 2.39, 95% CI [0.031, 0.307]). Taken together, the meta‐analytic results indicate that grandiose narcissism is associated with a blunted ERN across analytic approaches, with larger effects for admiration than rivalry.

## 5 General Discussion

An outstanding question in the narcissism literature concerns the neural processes underlying this personality trait (Sedikides 2021a). The literature has concentrated mainly on self‐reports, but the neural underpinnings of narcissists' thoughts, feelings, and behaviors are largely unknown. To address this gap, we assessed the relation between grandiose agentic narcissism and neural responsivity to errors, testing competing hypotheses. Whereas the mask model (Freud 1957; Kernberg 1975; Kohut 1966) posits that narcissists have a fragile core, the metacognitive model (Carlson 2013; Carlson et al. 2011; Podsiadłowski et al. 2025) postulates that this core is robust and well‐guarded through error denial. These contrasting perspectives on the narcissistic core led us to derive different hypotheses about early (< 100 ms) neural responses to errors. The mask and metacognitive models make opposing predictions regarding the ERN (i.e., enhanced vs. blunted, respectively), so results in either direction would help to distinguish between these two theoretical perspectives.

Across two studies and an internal meta‐analysis, grandiose agentic narcissism was associated with a blunted ERN, lending support to the metacognitive model. These findings should be viewed as early evidence rather than definitive conclusions: the samples were modest, the effects were small to moderate, and several analyses had limited power. Although we addressed these issues through internal reliability checks, Bayesian analyses, and supplementary analyses using more permissive inclusion thresholds, the present work marks an initial step in mapping error‐related neural processes onto narcissism. Larger and more diverse samples will be needed to refine effect‐size estimates, clarify boundary conditions, and test the stability of these associations across tasks and contexts.

### 5.1 Implications

Only one prior investigation, conducted by Mück et al. (2023), has examined error‐related brain activity in the context of narcissism. First, participants filled out the NARQ. Then, they completed a Go/NoGo task: The first block of trials included no self‐threatening, whereas the second block of trials included self‐threatening, normative performance data, and percentile rankings. Rivalrous, but not admirative, narcissism was associated with an elevated (more negative) ERN. However, this result may have been partly due to the increased cognitive accessibility of the construct “narcissism” (Skowronski et al. 2010), thus amplifying brain responses to the subsequent self‐threatening feedback. In addition, prior work has shown that narcissists will improve their performance and willingness to complete difficult tasks post negative (but not post neutral) feedback (Nevicka et al. 2016); as such, the direct threat of negative perception may alter their processing of performance and thus their error monitoring. Relatedly, narcissists will withdraw effort when opportunities for self‐enhancement are unavailable during a dart throwing task (Roberts et al. 2019), supporting the idea that they only monitor errors (i.e., exert cognitive resources) when threatened. Moreover, the Mück et al. sample size was relatively small. Finally, the Mück et al. results may reflect reduced ERN reliability. Consistent with prior work (Olvet and Hajcak 2009) and the current findings, a minimum of six‐error trials is needed to obtain a reliable ERN. Yet, as shown in table 2 of Mück et al., some participants conducted as few as four errors, potentially compromising the robustness of their neural estimates. In our research, participants completed the NARQ (embedded in a battery of other measures) at the end of the study session, eliminating possible priming influences. Moreover, we tested a larger sample and verified the reliability of the ERN.

Alternatively, our results complement those of the abovementioned pioneering investigation (Mück et al. 2023). Given that narcissists insulate themselves from self‐threats (Howes et al. 2020; Littrell et al. 2024) and disregard feedback (Kernis and Sun 1994; Kausel et al. 2015; O'Reilly and Hall 2020), their self‐views will remain positive (John and Robins 1994; Krizan and Herlache 2018; Sedikides and Campbell 2017), contributing to a blunted ERN at baseline. However, when explicitly and unavoidably confronted with self‐threatening feedback, rivalrous (but not necessarily admirative) narcissists may evince an elevated ERN. This possibility aligns with behavioral evidence (Hardaker et al. 2021; Horvath and Morf 2009) indicating that narcissists exhibit hypervigilance to self‐threatening stimuli—reflected in faster reaction times—when their psychological fragility is momentarily exposed. Although these studies did not employ neural measures, they suggest that task manipulations that heighten the salience of self‐threat can shift narcissists' information processing strategies. Future research should integrate these behavioral findings (Hardaker et al. 2021; Horvath and Morf 2009) with the neural evidence from the current studies and Mück et al. (2023) to clarify how self‐threat modulates error monitoring in narcissism.

Another difference between Mück et al. (2023) and our research is the task used to elicit the ERN. Mück and colleagues employed a Go/NoGo task, whereas we used a flanker task. Prior work suggests that the ERN is a stronger predictor of behavioral adjustments, such as next‐trial accuracy and reaction time, in tasks like the flanker and Stroop, but not consistently in the Go/NoGo task (Park et al. 2024). Mück et al. (table 4) reported no association between the number of errors and ERN amplitude (see their table 4). In contrast, we found that a greater number of errors was associated with a smaller (less negative) ERN in Study 1. Although this association did not replicate in Study 2, the observed pattern supports the possibility that the flanker task is more sensitive than the Go/NoGo task to individual differences in error‐related brain activity, including those related to narcissism. For these reasons, the flanker task is likely better suited than the Go/NoGo task to track narcissism. Consistent with this possibility, the flanker task is preferable for studies linking the ERN to individual differences because it achieves high reliability with few errors and with ERN magnitudes remaining stable within individuals and across increasing error trials (Meyer et al. 2013). Nonetheless, the discrepancy between the results of the prior investigation (Mück et al.) and ours may be due to different cognitive control strategies being implemented on the Go/NoGo task as compared with both the flanker and Stroop tasks. In the Go/NoGo task, participants must refrain from responding on all NoGo trials. In contrast, the Stroop and flanker tasks require a response on every trial but require the selective inhibition of visual distractor elements on only some trials (Meyer and Hajcak 2019; Park et al. 2024). Hence, different cognitive control strategies may account for the divergent ERN‐narcissism relations observed. Future research could test this hypothesis.

Our findings have implications for understanding the functional role of the ACC. The ERN and Reward Positivity are thought to reflect cognitive control and reinforcement learning functions of the ACC, respectively. Over the last three decades, one of the most debated topics in cognitive neuroscience concerns the ACC functions (Holroyd and Verguts 2021). The findings contribute to this debate by highlighting the role of motivational processes (i.e., self‐enhancement, self‐protection; Alicke and Sedikides 2009; Sedikides 2021b) in the cognitive control functions of the ACC. Given that Reward Positivity is also generated in part by the ACC, a lingering question concerns the relation between narcissism and the Reward Positivity. If grandiose agentic narcissism relates to the Reward Positivity in the same way it does to the ERN, then grandiose agentic narcissism might produce a broader deficit in feedback processing that includes deceits in both cognitive control and reinforcement learning. Such a neurocognitive model would lay the foundation for research linking neural markers of error and feedback processing to the intrapersonal (e.g., feelings of superiority, internalizing positive feedback, derogating the sources of negative feedback) and interpersonal (e.g., looking down on others, seeking adoration from others) strategies that narcissists use to regulate their self‐esteem (Morf and Rhodewalt 2001). The links between these neural markers and self‐esteem regulation may advance understanding of the adversarial character of narcissists' interpersonal relationships as colleagues, friends, partners, and leaders. Follow‐up research could consider these directions.

### 5.2 Limitations and Future Empirical Paths

The present findings offer an initial demonstration of a link between narcissism and blunted error‐related brain activity, but they remain preliminary and should be interpreted in that light. Despite the vast literature on narcissism, there is a dearth of studies examining its neural underpinnings. The majority of those, including our investigation, have examined grandiose agentic narcissism. This form of narcissism is characterized by self‐enhancement on agentic dimensions, extolling one's own competence, decisiveness, creativity, intelligence, ambition, and vision. Following in this tradition, we focused on grandiose agentic narcissism, and in particular admiration and rivalry. Narcissism, though, comprises other forms (Sedikides 2021a): vulnerable, communal (characterized by self‐enhancement on communal dimensions—extolling one's own warmth, pro‐sociality, morality, and holiness), and collective (egocentric exceptionalism of one's ingroup). Does the blunted ERN we observed extend to these additional forms of narcissism or is it more uniquely associated with grandiose agentic narcissism? Further, does the egocentric exceptionalism that collective narcissists feel for their ingroup manifest in a blunted ERN when ingroup members make mistakes? These questions merit empirical attention.

Follow‐up research should also address psychopathological correlates and additional neural markers that may illuminate the complex, multifaceted nature of narcissism at the physiological micro‐level. Given the established association between narcissism and various forms of psychopathology, along with the conceptual overlap with the externalizing spectrum whose traits and disorders have likewise been linked to reduced ERN, it is crucial to determine whether the observed effects are unique to narcissism or reflect general externalizing processes. Indeed, studies show a diminished ERN as a robust neural correlate of externalizing behaviors, including substance abuse (Steele et al. 2014), antisocial behavior (Lutz et al. 2021), impulsivity (Hall et al. 2007), and psychopathy (Von Borries et al. 2010). This association is also consistent with externalizing disorders that overlap with narcissism like aggression (Sun et al. 2023) and social pain following exclusion (Themanson et al. 2014). As such, reduced error processing may represent a more generalized externalizing liability rather than a marker for a specific disorder or process. Therefore, the current findings may offer further support for this broader pattern, with narcissism reflecting one expression of externalizing vulnerability. Clarifying these associations will advance the burgeoning field of personality neuroscience, and in particular the development of robust neuroscientific models that explain the biological basis of variation in human personality (Corr and Mobbs 2018).

There is disagreement about the extent to which conscious error awareness contributes to the ERN. Some suggest that the ERN reflects features of error processing below the surface of consciousness awareness, with conscious error awareness captured by a later ERP, the error positivity (Overbeek et al. 2005). However, others propose that error awareness is related to the ERN (Di Gregorio et al. 2018, 2020, 2022; Scheffers and Coles 2000; Wessel et al. 2011). Thus, an open issue for future research concerns the role of conscious awareness in the link between narcissism and error‐related brain activity. Studies that experimentally manipulate error awareness found that it reflects post‐response conflict rather than error awareness (Di Gregorio et al. 2016). Thus, grandiose agentic narcissists experience less post‐response conflict after errors rather than being less consciously aware of their errors. This interpretation is consistent with the reported null relation between grandiose agentic narcissism and error positivity (Mück et al. 2023). Further, the null relation is compatible with behavioral evidence that grandiose agentic narcissists experience less conflict (i.e., rely on intuition and ignore experts), clouding their decision‐making (O'Reilly and Hall 2020). Future neuroscientific inquiry that directly manipulates error awareness and tracks narcissists' decision‐making in real time is needed.

Another open question concerns the oscillatory dynamics that give rise to the ERN. The ERN has been closely linked to frontal midline theta oscillations, which represent the engagement of cognitive control during error processing (Cavanagh et al. 2009; Keil et al. 2010; Luu et al. 2004; Trujillo and Allen 2007). Although we did not assess oscillatory activity, Zhou et al. (2025) demonstrated that distinct narcissism facets are reflected in spontaneous resting‐state oscillations, suggesting that narcissism may be underpinned by characteristic oscillatory patterns. Future investigations should integrate these literatures by examining whether task‐evoked theta power mediates the link between narcissism and error‐related brain activity. Such work would clarify whether the blunted ERN observed in grandiose agentic narcissists reflects reduced recruitment of theta‐based cognitive control processes during error monitoring.

Our participants were predominantly white, British University students. Thus, the generality of the findings is constrained both demographically and culturally. Rooted in Greek mythology, narcissism was presumed by some to be a Western phenomenon (Foster et al. 2003), but the picture may be more nuanced. For example, a study involving English‐speaking adults from across the globe assessed grandiose agentic narcissism with the Narcissistic Personality Inventory (Raskin and Terry 1988) and found that the Leadership/Authority and Grandiose Exhibitionism facets of the Narcissistic Personality Inventory were culturally invariant, whereas the Entitlement/Exploitativeness facet was not. Further, individuals from more collectivistic cultures reported higher levels of Entitlement or Exploitativeness than those from individualistic cultures (Fatfouta et al. 2021). Likewise, another study (Jauk et al. 2021) found higher levels of entitlement in Japan (a collectivist culture) than Germany (an individualistic culture). Thus, narcissism is not a uniquely Western construct, and some facets may be elevated in Eastern cultures. Along these lines, there is evidence that the ERN is modulated by cultural context (Sedikides et al. 2024). Face priming enhanced the ERN for East‐Asian participants but decreased it for European–American participants (Park and Kitayama 2014). The researchers proposed that these contrasting patterns stem from cultural differences in self‐perception. East‐Asians, who typically have an interdependent self‐construal, may find facial images more self‐threatening. In contrast, European–Americans, who typically have an independent self‐construal, may find facial images less self‐threatening. Given the nuanced cultural variability of both narcissism and the ERN, future research may examine whether a blunted ERN is associated with elevated grandiose agentic narcissism across cultures and contexts. The nature of these associations might be contingent on facets of grandiose agentic narcissism (e.g., Entitlement/Exploitativeness) or aspects of culture (e.g., individualism/collectivism).

Finally, despite our efforts to increase sample size relative to prior research, sensitivity analyses in Study 1 suggested that power was still somewhat limited, particularly for detecting associations with the ΔERN. To address this, we conducted a replication and extension in Study 2 using a similar task that included explicit performance feedback, and we complemented this practice with an internal meta‐analysis across the two studies. This approach yielded more precise estimates and showed that the associations, though modest in size, were consistent and reliable across analytic methods.

Relatedly, across both studies, some analyses were appropriately powered whereas others were not, based on sensitivity analyses. In Study 1, the bivariate correlations were slightly underpowered, but the analyses controlling for the number of errors were sufficiently powered to detect the observed association between overall narcissism and the residual ERN. In Study 2, analyses involving admirative narcissism were appropriately powered, and analyses controlling for the number of errors were sufficiently powered to detect the observed associations between overall narcissism and both the ERN and the residual ERN. To complement these frequentist analyses, we conducted Bayesian tests for all focal associations in both studies. These yielded anecdotal to moderate evidence in favor of the observed effects, providing an additional check on evidential strength under limited sample size. In both studies, the effects were modest but consistent in direction and interpretation. Future work with larger and more diverse samples will be important for refining effect‐size estimates and establishing generalizability, but the present findings nevertheless offer valuable preliminary evidence under rigorous methodological control.

A further limitation concerns the exclusion criteria applied to participants with insufficient error trials. In Study 2, we excluded 28 participants because they did not commit any errors and thus could not be part of analyses on error‐related processes. This exclusion reduced the sample size, but it was necessary to ensure the validity of the ERN. The ERN is a sensitive measure that demonstrates acceptable reliability only when a minimum number of error trials is available, typically at least six (Meyer et al. 2013; Olvet and Hajcak 2009; Pontifex et al. 2010), with some research suggesting an even higher threshold (Larson et al. 2010). We next conducted internal reliability analyses, which indicated that six errors were sufficient in Study 1 and two in Study 2. We adopted the stricter six‐error threshold across studies. Although this practice led to the exclusion of additional participants in Study 2, it conferred a more reliable ERN and maintained consistency across studies.

### 5.3 Conclusion

Our research lays the groundwork for a more precise neuroscientific account of narcissism and raises promising questions. Is narcissism conducive to changes in neural processes? Do neural processes scaffold the development and maintenance of narcissism? Do attenuated error or feedback processing underlie the intra‐ and interpersonal consequences of narcissists' self‐regulation strategies? Addressing these questions may deepen understanding and inform efforts to reduce the interpersonal and societal costs of narcissism.

## Author Contributions

**Esther M. Robins:** formal analysis, investigation, methodology, software, visualization, writing – original draft. **Zhiwei Zhou:** investigation, formal analysis, writing – review and editing. **Chengli Huang:** investigation, writing – review and editing. **Douglas J. Angus:** conceptualization, methodology, writing – review and editing. **Constantine Sedikides:** conceptualization, supervision, writing – review and editing. **Nicholas J. Kelley:** conceptualization, project administration, supervision, writing – review and editing.

## Funding

The authors have nothing to report.

## Conflicts of Interest

The authors declare no conflicts of interest.

## Data Availability

The data that support the findings of this study are openly available in OSF at https://osf.io/gubyp/.

## Endnotes

^1^
Individuals toward either end of the distribution are often labeled “narcissists” or “non‐narcissists.” We occasionally use this terminology for simplicity. We treat narcissism dimensionally in all data analyses.

^2^
Narcissism scores (total, admiration, rivalry) did not significantly differ between participants who made no errors and those who made at least one error, *ts* < 1.17, *p*s > 0.245.

^3^
To ensure transparency, we conducted ancillary analyses with more permissive error‐count thresholds in Study 2. Although 28 participants made no errors and could not be included under any criterion, lowering the threshold to two errors (*N* = 77) or one error (*N* = 81) yielded the same result pattern. Admirative narcissism continued to predict a blunted ERN (*r* = .27, *p* = 0.020), and the association with overall narcissism was similar in magnitude to Study 1's (*r* = .20, *p* = 0.06).

^4^
Following the Flanker Task and prior to completing the questionnaire, participants performed a Stroop task and an incentive delay task, both of which were unrelated to the current hypotheses.
